# The effectiveness of interventions for optometric referrals into the hospital eye service: A review

**DOI:** 10.1111/opo.13219

**Published:** 2023-08-25

**Authors:** Josie Carmichael, Sarah Abdi, Konstantinos Balaskas, Enrico Costanza, Ann Blandford

**Affiliations:** ^1^ University College London Interaction Centre (UCLIC), UCL London UK; ^2^ NIHR Biomedical Research Centre at Moorfields Eye Hospital NHS Foundation Trust and UCL Institute of Ophthalmology London UK

**Keywords:** enhanced referrals, false‐positive, independent prescribing, optometrists, optometry, primary eye care, referrals, teleophthalmology

## Abstract

**Purpose:**

Ophthalmic services are currently under considerable stress; in the UK, ophthalmology departments have the highest number of outpatient appointments of any department within the National Health Service. Recognising the need for intervention, several approaches have been trialled to tackle the high numbers of false‐positive referrals initiated in primary care and seen face to face within the hospital eye service (HES). In this mixed‐methods narrative synthesis, we explored interventions based on their clinical impact, cost and acceptability to determine whether they are clinically effective, safe and sustainable. A systematic literature search of PubMed, MEDLINE and CINAHL, guided by the Preferred Reporting Items for Systematic Reviews and Meta‐Analyses (PRISMA), was used to identify appropriate studies published between December 2001 and December 2022.

**Recent Findings:**

A total of 55 studies were reviewed. Four main interventions were assessed, where two studies covered more than one type: training and guidelines (*n* = 8), referral filtering schemes (*n* = 32), asynchronous teleophthalmology (*n* = 13) and synchronous teleophthalmology (*n* = 5). All four approaches demonstrated effectiveness for reducing false‐positive referrals to the HES. There was sufficient evidence for stakeholder acceptance and cost‐effectiveness of referral filtering schemes; however, cost comparisons involved assumptions. Referral filtering and asynchronous teleophthalmology reported moderate levels of false‐negative cases (2%–20%), defined as discharged patients requiring HES monitoring.

**Summary:**

The effectiveness of interventions varied depending on which outcome and stakeholder was considered. More studies are required to explore stakeholder opinions around all interventions. In order to maximise clinical safety, it may be appropriate to combine more than one approach, such as referral filtering schemes with virtual review of discharged patients to assess the rate of false‐negative cases. The implementation of a successful intervention is more complex than a ‘one‐size‐fits‐all’ approach and there is potential space for newer types of interventions, such as artificial intelligence clinical support systems within the referral pathway.

**Key points**

- Several approaches have been implemented to address inappropriate optometric referrals seen in face‐to‐face hospital eye services, with all demonstrating clinical effectiveness.
- The literature suggests stakeholder acceptance of referral filtering schemes; however, evidence for acceptance of other interventions is lacking. More studies are required to assess the cost‐effectiveness and safety of all interventions.
- The success of interventions can vary depending on which outcome and stakeholder is considered, meaning there is no ‘one‐size‐fits‐all’ approach.

## INTRODUCTION

In the UK, most referrals to the hospital eye services (HES) originate from primary care optometric examinations. One study carried out in Bradford, UK, found that 72% of all referrals into the HES originated from optometrists,^1^ with the remaining referrals initiated by general medical practitioners. The General Optical Council (GOC) standards of practice guidelines state that optometrists should ‘recognise and work within the limits of their scope of practice’.^2^ Consequently, optometrists may act cautiously when unsure about diagnoses and refer patients into the HES unnecessarily, creating ‘false‐positive’ referrals^3^, ^4^ that contribute to demand on an already over‐burdened HES. In a quantitative systematic review of optometrists' referral accuracy and the contributing factors, we found significant variation in reported referral accuracy both within and across different ocular conditions.^5^

Recognising the need for intervention, several approaches have been trialled to tackle the high numbers of referrals. For example, in glaucoma care, referral filtering schemes have been implemented to ‘triage’ low‐risk patients by optometrists with higher training and certification^6^ through repeating, enhancing or refining the findings from the community eye examination before deciding whether onward referral to the HES is appropriate. More recently, with the advancement of ocular imaging, there has also been a focus on the implementation of teleophthalmology services for asynchronous referral review and triage, which has been shown to reduce the number of unnecessary referrals for retinal disease from entering the HES.^7^, ^8^ Furthermore, the significant surge in the development of artificial intelligence (AI) for medical imaging^9^, ^10^ has highlighted a potential for its use in a range of applications including eye care. Of course, these AI systems require rigorous evaluation before implementation.

In this narrative review, we aimed to explore the literature for interventions that have been implemented or piloted to reduce the number of false‐positive referrals entering face‐to‐face clinics in the HES. We aimed to use our findings to determine aspects of each approach that have been successful or unsuccessful and to get an overview of which approaches were being focussed on in different areas within the UK and globally.

## OBJECTIVES

The review aimed to address the following specific questions:

1. What approaches have been made to try and reduce the number of false‐positive referrals seen in face‐to‐face HES clinics?
2. How successful have these approaches been in reducing the number of false‐positive referrals seen in the HES?
3. Are these approaches sustainable? That is, are they cost‐effective, safe and accepted by stakeholders?

## METHODS

### Registration

The international prospective register of systematic reviews (PROSPERO) was used to register our review protocol (registration number: CRD42022328773) in order to prevent review duplication and increase the transparency of our review process.

### Eligibility criteria

In order to complete a robust systematic search and selection of studies, a checklist of inclusion and exclusion criteria was created. This was to ensure consistency when screening articles and to act as a reference point when making decisions about whether to include/exclude articles. The decision was made to exclude studies that assessed diabetic screening referrals because, although many optometrists work as diabetic screening graders and make referral decisions, this pathway does not represent the typical primary care referral pathway. Table 1 summarises the inclusion and exclusion criteria.

**TABLE 1:** Summary of the inclusion/exclusion criteria.

Criteria	Inclusion	Exclusion
Time period	Dec 2001–Dec 2022	Prior to Dec 2001
Language of original study	English	Any other language
Study design	Qualitative, quantitative and mixed‐methods design including (but not limited to): controlled, uncontrolled studies, observations, interviews, surveys, retrospective analysis, clinical vignettes	Viewpoints, editorials, conference/meeting abstracts, expert opinions and grey literature. Systematic or similar reviews (e.g., narrative, scoping and realist reviews)
Setting	Any setting involving primary eye care	Secondary care internal referrals, GP referrals, self‐referrals, referrals from a diabetic retinopathy screening programme
Participants	Studies focussing only on primary care optometrists making referrals to secondary care	Studies focussing on referrals from GPs, diabetic retinopathy screening programmes, other allied health professionals or patients who self‐refer (e.g., patients attending Accident and Emergency without the recommendation from an optometrist)
Condition focus	Any eye condition or conditions (can include anterior and posterior eye conditions)	Referrals by optometrists to non‐HES departments due to systemic conditions showing signs in the eye (e.g., referral to GP for blood pressure check due to mild hypertensive retinopathy)
Topic focus	Interventions that have been implemented, trialled or piloted. Studies do not just need to focus on the clinical outcome of these interventions. They may focus on other measures of effectiveness Interventions can take place anywhere along the referral pathway before patients are seen face to face in the HES	Programmes or schemes that have been implemented to improve referral system but not to reduce or triage referrals into the HES

Abbreviations: GP, general medical practitioner; HES, hospital eye service.

We included primary studies that used a quantitative, qualitative or mixed‐methods design and were written in English. We did not exclude studies based on our assessment of methodological limitations, as described below, but used the information about methodological limitations to assess our confidence in the findings. We excluded abstracts without a corresponding full paper, as they were unlikely to provide sufficiently rich data.

### Search strategy

The PRISMA guidelines were used to guide our protocol development.^11^ PubMed, MEDLINE and CINAHL were searched for potential studies for inclusion. Initially, a search was also performed using Google Scholar, but this returned many irrelevant results, with relevant papers being duplicated from the other databases. We developed search strategies for the databases. Studies published during or after December 2001 were included to ensure an assessment that is representative of recent practice. Table 2 presents the final facets and keywords used when searching databases. In addition to database searching, we reviewed the reference lists of all included studies and other key references which allowed a method of ‘reference chaining’.

**TABLE 2:** Facet terms and their keywords used for database searching.

Number assigned to facet	Facet	Keywords	Boolean
1	Optometrist	Optometrist(s) OR Optometry OR Primary eye care OR Primary eye clinic(s) OR Optician(s)	1 AND 2
2	Referral practice	Referral(s)	

### Selection process

All articles identified from database searches were organised in EndNote and duplicates were removed. The primary researcher (JC) conducted screening of the titles and abstracts of all search results. A second researcher (SA) also screened all titles and abstracts. Initially, a sample of 20% was screened by both the researchers to assess agreement. All articles where the researchers disagreed were reviewed together and differences in interpretation of the inclusion/exclusion criteria were discussed at this stage. The remaining studies (80%) were screened by both researchers independently with a good level of agreement (κ = 0.84; 95% CI: 0.77–0.90). Studies where the two reviewers disagreed were discussed and a decision was reached to include/exclude each one. After the screening phase, 111 studies met the criteria for full‐text assessment.

The full texts of all 111 studies were assessed by the primary researcher. The secondary researcher screened the full text for a sample of 20% (22 studies) and agreement was checked. Due to a small sample size, kappa agreement could not be calculated. There was 90.9% (20/22) agreement between the two reviewers. For two studies, the reviewers initially disagreed, but after discussion based on the inclusion/exclusion criteria, they agreed that both studies should be excluded.

### Data collection and items

Data collection was carried out by one reviewer (JC) who worked independently. Prior to collection, a form was designed to extract all relevant data from each included study. This form was part of a study protocol, which was written by JC and reviewed by SA and AB prior to data extraction. Table 3 summarises the information extracted from each article.

**TABLE 3:** Information extracted from all studies included in the review.

Information extracted	
1	Author(s)
2	Year
3	Title
4	Country
5	Study aim(s)
6	Study design
7	Sample period
8	Sample size
9	Eye condition(s)
10	Type of intervention
11	Main results
12	Limitations
13	Other important findings

### Quality assessment

In this review, we focussed on papers which were the most relevant, rather than papers which met a specific standard of methodological quality. This has previously been described as prioritising ‘signal’ over ‘noise’.^12^ Rather than excluding studies based on quality, they were included but critiqued during review to ensure transparency.^13^ When critiquing study quality, we mainly focussed on sample size for referrals, number of optometrists from which the referrals originated, number of practices from which the referrals originated, study design with respect to prospective or retrospective analysis and the appropriateness of any statistical methods that were used.

### Synthesis of results

A narrative synthesis approach^14^ was taken when reporting the results. This was chosen as we wanted to provide a detailed assessment of studies into different clinical interventions, while keeping an exploratory approach. We aimed to keep our research question broad with respect to study focus and definitions used across the studies; however, we recognise that the review is more aggregative than interpretive. We summarised the results with respect to types of interventions and the outcomes assessed. We referred to the Economic and Social Research Council (ESRC) guidance on the conduct of narrative syntheses^15^ when carrying out this review to increase transparency and trustworthiness. The framework consisted of four elements:

1. Developing theory of how the intervention works, why and for whom.
2. Developing a preliminary synthesis of findings of included studies.
3. Exploring relationships within and between studies.
4. Assessing the robustness of the synthesis.

## RESULTS

### Study selection

**FIGURE 1:**
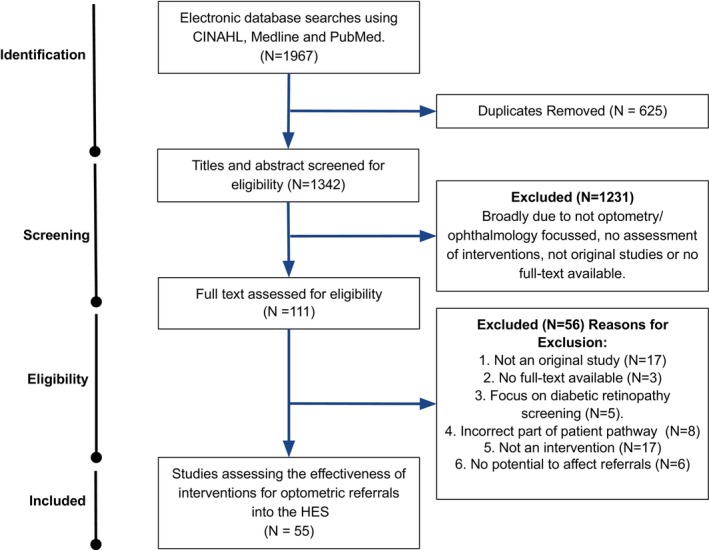
PRISMA flow chart detailing the selection process for the studies reviewed.

Fifty‐five studies were selected for analysis. The results from the search and selection process are shown in Figure 1.

### Study characteristics

**FIGURE 2:**
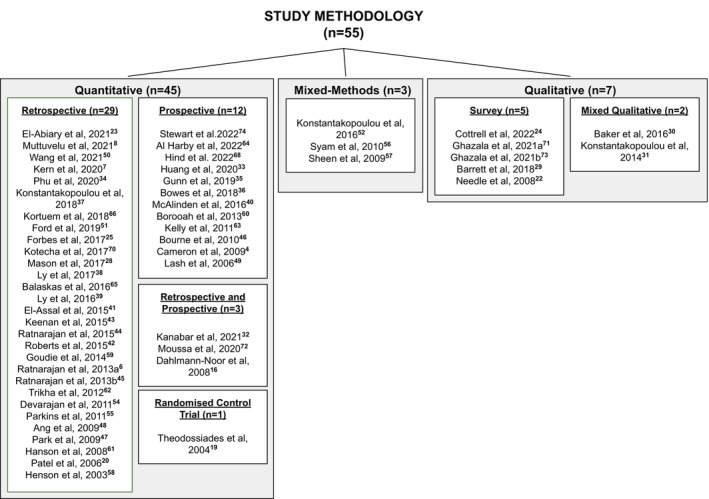
An overview of the methodology used in each of the 55 studies reviewed.

Details of the 55 reviewed study designs can be found in Figure 2 and in the Supporting Information. When reviewing the literature, it was clear that there were several different interventions that had been implemented or piloted to improve the accuracy of referrals into the HES. These interventions could be categorised into four groups:

1. Training and guidelines
2. Referral filtering schemes
3. Asynchronous teleophthalmology
4. Synchronous teleophthalmology

Some studies used multiple approaches and were therefore included in more than one type of intervention category. In this section, we discuss the outcomes reported within these four groups and consider their success indicators for both reducing false‐positive referrals seen face to face in the HES and determining their safety and sustainability.

### Training and guidelines

One approach to improve the accuracy of referrals entering the HES was to focus on improving the skills and knowledge of community optometrists as the main source of ophthalmic referrals from primary care^16^ and/or introducing clear clinical guidelines that can be followed when making referral decisions. A summary of the studies focussing on this approach can be found in Table S1.

The most recent of these studies^17^ assessed the impact of implementing clear referral guidelines set out by the Scottish Intercollegiate Guidelines Network (SIGN).^18^ These provide guidance on the primary care assessment of patients with suspected glaucoma and clear referral criteria for optometrists practising in Scotland. Following the publication of these new guidelines, this particular study reported a significant decrease in HES glaucoma clinic first discharge rates from 29.2% to 19.4% (*p* = 0.004) due to a lower proportion of patients being referred unnecessarily to clinics.

Two studies carried out in England assessed the impact of formal training sessions on the accuracy of glaucoma referrals. Theodossiades et al.^19^ focussed on training in optic nerve evaluation as well as providing referral criteria. The authors reported that the proportion of referrals from the intervention group resulting in a positive outcome (positive predictive value [PPV] = 0.49) was very similar to that of the control group (PPV = 0.46). A follow‐up from this study,^20^ which assessed the impact of ongoing training every 4 months, found that the training resulted in a 58% increase in the number of referrals compared to the original study; however, the PPV remained very similar (PPV = 0.51). Thus, for these two studies, participants appeared to have been detecting more true‐positive cases, but they had not improved their skills for confidently ruling out glaucoma in patients without the disease. Glaucoma suspects are encountered infrequently in primary care practice, meaning it is difficult for optometrists to confidently rule out the disease, particularly in its early stages. Its characteristically progressive nature means that even within the HES, more than one follow‐up visit may be required before patients are determined to not have the disease.^21^

For training and guidelines to be deemed successful interventions, they must also be sustainable. We found no literature addressing the cost‐effectiveness of the training described. There were, however, studies addressing optometrists' uptake and opinions towards further training. In a survey study published in 2008,^22^ assessing optometrists' opinions on the Department of Health's announcement that with suitable qualification, optometrists will be able to train as independent prescribers (IPs), only 9% reported no intention of undergoing further training for prescribing. However, optometrists expressed concerns such as a lack of time for training being a substantial barrier for 64% of respondents. Although that study is now dated, the findings may partially explain why a more recent study^23^ found that less than a quarter (23.4%) of optometrists hold an IP qualification in Scotland. Barriers to extra training must be considered when implementing training programmes for primary care optometrists to maximise uptake, especially since health boards in Wales with IP optometrist commissioned services had fewer total and urgent referrals to ophthalmology compared with health boards with no IP optometrists during 2020.^24^ However, as the study reporting these findings took place in 2020, during the COVID pandemic, the results may not truly represent the demographics of patients usually presenting to primary care services.

### Referral filtering schemes

Another approach that has been adopted in the UK, as well as in other countries, to improve referral accuracy, is to introduce referral filtering schemes. These schemes also utilise the interventions of training and guidelines but specifically for funded pathways where optometrists perform additional testing and assessment and act as a triage for low‐risk patients. For glaucoma, there are three types of filtering schemes that have been implemented: repeat measures where intraocular pressure and/or visual fields are repeated prior to making a referral decision, enhanced case finding where optometrists undertake a higher level of assessment compared to repeating measures and finally, glaucoma referral refinement which offers a level of testing by certified optometrists which is sufficient for glaucoma diagnosis.^25^ Previous reviews have assessed the effectiveness of these individual schemes.^26^, ^27^ We aimed to update the literature in this area as well as compare these schemes to other types of interventions. Our review identified many studies which met our inclusion criteria (*n* = 32) and focussed on this approach. Due to this large number, we display a summary of the studies for this type of intervention, grouped by the factor(s) focussed on when assessing the scheme (Table 4).

**TABLE 4:** Summary of studies focussing on referral filtering schemes, grouped based on outcomes assessed.

Focus	Author(s)	Year	Location
Cost assessment	Forbes et al.^25^	2019	UK
	Mason et al.^28^	2017	UK
Acceptability	Barrett and Loughman^29^	2018	Ireland
	Baker et al.^30^	2016	UK
	Konstantakopoulou et al.^31^	2014	UK
Clinical impact	Kanabar et al.^32^	2021	UK
	Huang et al.^33^	2020	Australia
	Phu et al.^34^	2020	Australia
	Gunn et al.^35^	2019	UK
	Bowes et al.^36^	2018	UK
	Konstantakopoulou et al.^37^	2018	UK
	Ly et al.^38^	2017	Australia
	Ly et al.^39^	2016	Australia
	McAlinden et al.^40^	2016	UK
	El‐Assal et al.^41^	2015	UK
	Roberts et al.^42^	2015	UK
	Keenan et al.^43^	2015	UK
	Ratnarajan et al.^44^	2015	UK
	Ratnarajan et al.^45^	2013a	UK
	Bourne et al.^46^	2010	UK
	Park et al.^47^	2009	UK
	Ang et al.^48^	2009	UK
	Lash et al.^49^	2006	UK
Mixed focus	Wang et al.^50^	2021	Australia
	Ford et al.^51^	2019	Australia
	Konstantakopoulou et al.^52^	2016	UK
	Ratnarajan et al.^53^	2013b	UK
	Devarajan et al.^54^	2011	UK
	Parkins and Edgar^55^	2011	UK
	Syam et al.^56^	2010	UK
	Sheen et al.^57^	2009	UK
	Henson et al.^58^	2003	UK

### Clinical impact

The evidence suggests that referral filtering schemes are clinically effective for triaging and managing patients that do not require HES review. Studies have reported that between 35% and 71% of patients were discharged after their first assessment within the schemes for glaucoma referral filtering and therefore not referred on to the HES.^43^, ^44^, ^46^, ^55^, ^58^ In Scotland, false‐positive glaucoma referrals significantly reduced (36.6%–21.7%, *p* = 0.006) following a new General Ophthalmic Services (GOS) contract in 2006 which funded community optometrists to perform supplementary examinations in glaucoma cases,^48^ with a later study^41^ supporting these findings.

Four UK studies reported the outcomes of patients seen as part of a scheme set up for patients with recently occurring minor eye conditions service (MECS). These investigations reported that between 66% and 75.3% of patients were managed by their optometrist without referral, either through first visit discharge or follow‐up by their optometrist,^37^, ^40^, ^52^, ^57^ and only 15.9%–18.9% were referred to the HES.^37^, ^40^, ^57^ In 2020, the COVID‐19 Urgent Eyecare Service (CUES) system, whereby initial screening took place via a telephone appointment by an optometrist, was adopted to allow HES clinicians to focus on more urgent eye care cases as recommended by CUES nationally in April 2020. In Manchester, this system resulted in only 13.0%–14.3% of cases being provisionally referred to secondary care HES.^32^ Four studies assessed the outcomes from patients seen in an Australian Centre for Eye Health (CFEH) set up as an intra‐professional, optometry‐led collaborative eye care clinic to triage patients referred for non‐urgent conditions. These investigations reported recommendation for referral to the HES in just 12%–16.3% of patients depending on the eye condition,^38^, ^39^, ^50^ and that 10.6 weeks of outpatient appointments were saved by assessing patients off‐site at the CFEH.^51^

Referral filtering schemes have also been developed for cataract referrals. These direct pathways have been introduced to ensure that the ‘Action on Cataracts’ guidelines were followed, and that patients referred for cataract surgery were only seen within the HES when they had reduced measured vision, were symptomatic and expressed a willingness for surgery. Two early^47^, ^49^ and one more recent study^36^ reported that surgery listing rates were significantly higher (83%–87%) when compared to conventional referral pathways (63%–78%).

It is clear from these findings that these schemes can successfully result in a reduced number of patients being seen in the HES unnecessarily. One important clinical factor to consider, however, is the possible resultant false‐negative cases. Of the studies reviewed, five assessed the false‐negative rate of patients,^35^, ^43^, ^44^, ^51^, ^54^ all of which assessed referral filtering schemes for glaucoma. These studies reported a false‐negative rate of between 2% and 15%, when reviewed either virtually or face to face. To improve clinical safety, some studies added an element of HES virtual review of all discharged patients as a failsafe. However, this required additional costs and resources.^44^, ^54^

### Cost assessment

In two Australian studies, a decrease in average cost per patient^51^ and no apparent change in cost^50^ were reported when using a newer referral refinement scheme compared to the standard pathway.

For the studies carried out in the UK, two reported cost saving of the MECS^28^, ^57^ and two for a glaucoma filtering scheme.^53^, ^54^ One evaluation compared two glaucoma schemes and reported a higher saving (62%) of a repeated measures scheme compared to enhanced referral refinement (3.5%).^55^ The last two studies reported results which differed depending on the assumption that was used for comparison.^58^ For example, a more recent investigation^25^ reported that while assuming there would be 2.3 outpatient visits to the HES avoided per person, the saving would be approximately £2.76 per patient passing through the scheme. However, when this assumption was changed to avoiding one appointment, there was an increase in costs of approximately £42.28 per patient. These findings highlight the difficulty of assessing cost‐effectiveness, as comparisons are usually based on assumptions and/or predictions.

### Acceptability

The reviewed studies suggest that there is an overall positive opinion from optometrists in relation to referral filtering schemes, which is essential as optometrists are required to play an active and engaged role. One recent study reported responses about the MECS scheme^31^ and focussed on reasons for optometrist participation, with the most common reason being for career development through experience of assessing challenging cases. Approximately 85% identified that training had a beneficial effect on their practice. Feedback from general medical practitioners and ophthalmologists was also supportive of the referral filtering schemes.^30^, ^31^ Studies reporting patient experiences with a referral filtering scheme were again positive overall.^57^ One study reported that all patients (*n* = 109) who completed a survey were satisfied, with 95% of the patients reporting confidence and trust in their optometrist.^52^

### Asynchronous teleophthalmology

Asynchronous review of clinical information has been used as a method of discharging patients. This method utilises a ‘store‐and‐forward’ approach of information uploading with review later. The benefit of these systems is that patients can receive a clinical opinion from a specialist HES clinician (ophthalmologist or optometrist) without having to be seen face to face. Five studies assessing systems using this approach used datasets from at least 10 years ago,^4^, ^59^, ^60^, ^61^, ^62^ whereby general ophthalmology or retinal referrals were sent by primary care optometrists with photographs attached. These studies all reported positive impacts on patient outcomes with 34%–48% reviewed virtually identified as not requiring referral for face‐to‐face review. This value increased to 80.5% in a more recent Danish study,^8^ perhaps due to improved quality of ocular imaging.

In order to improve the ability of clinicians to triage patients virtually, more information may be uploaded for review including advanced ocular imaging such as optical coherence tomography (OCT), which is now more widely available in primary care. Two studies included uploading of OCT images along with fundus photographs.^7^, ^63^ The more recent study from the UK^7^ assessed referrals, specifically for retinal conditions, and found that 52% of the patients classified into the referral pathway did not require specialist referral.

Technician‐delivered, hospital‐based clinics including ocular imaging have become another useful way to review new patient referrals.^64^, ^65^, ^66^ The successful upscaling of the virtual clinical capacity for glaucoma patients at Moorfields Eye Hospital now means that all new routinely referred patients (around 5000 per annum) can be seen virtually.^67^ In a pilot clinic design, a recent study reported substantial agreement between the diagnosis reached by clinicians reviewing patients with suspected lid lesions face to face than when photographs of the lesion were reviewed by consultants.^68^

It is clear from the results discussed that asynchronous review of referrals can successfully reduce the number of patients needing to be seen face to face in the HES. However, we must again consider whether this method of triage is safe and sustainable. For glaucoma diagnosis, the UK National Institute for Health and Care Excellence (NICE) guidelines recommend that patients undergo testing with traditional in‐person review, including standard automated perimetry, Goldmann applanation tonometry, anterior chamber angle assessment with gonioscopy and dilated optic nerve and fundus examination with slit lamp biomicroscopy,^69^ with the latter three tests not possible in a technician‐led virtual glaucoma clinic. Only one study assessed the false‐negative rate of the asynchronous referral scheme for glaucoma and found that 20% seen for a face‐to‐face appointment after being discharged virtually were determined to require HES review (4% of whom required medical intervention and were considered as ‘significant’ false negatives).^70^ Another study reported that 40% of patients were discharged without intervention from a clinic assessing eyelid lesions, whereas discharge was recommended in 51.6% for the same set of patients when reviewed virtually. Of note, the virtual reviews were performed by a separate consultant ophthalmologist in the latter study.^68^ In relation to the sustainability of these systems, no information about cost was reported and it was unclear from the literature how acceptable these systems were to the stakeholders using them. Just one study reported patients' opinions on being reviewed virtually, with only 3/114 (2.6%) patients stating that they preferred face‐to‐face review over virtual assessment.^4^

### Synchronous teleophthalmology

Virtual patient assessment via teleophthalmology is also possible synchronously, meaning that patients do not have to be seen face to face to be examined in real time. Synchronous teleophthalmology is not just useful to avoid in‐person contact with patients (particularly for safety reasons during the COVID‐19 pandemic) but can also be used to connect primary care optometrists to secondary care physicians during an examination.

Five studies focussed on the assessment of synchronous teleophthalmology services which were implemented in response to the COVID‐19 pandemic. One^71^ used a live platform for a range of different ophthalmic conditions and reported that pre‐lockdown using this system, 50/78 (64.1%) of referrals to secondary care had been avoided. During lockdown, this increased to 65/76 (85.5%). Another approach was using telephone triage.^72^ One study assessed a telephone triage service manned by HES allied healthcare professionals and ophthalmologists. Using this system, less than a quarter (24.5%) of patients required face‐to‐face follow‐up. In Greater Manchester in the UK, 38% of patients did not require a face‐to‐face appointment when using remote triaging as part of the CUES scheme.^32^

Although data were limited for stakeholder opinions, when considering synchronous video assessment the mean Likert score for satisfaction with a teleophthalmology consultation was 5/5 from optometrists, ophthalmologists and patients.^73^ Another investigation also reported that 98.5% of patients felt comfortable with the quality of a telemedicine examination, with 97.1% reporting they would participate in another one in the future.^74^ However, no studies reported the cost‐effectiveness of these systems or the logistics of having clinicians on call, in real time, to assess patients. Additionally, four studies were carried out during COVID‐19 lockdowns meaning fewer patients would have been visiting optometrists for routine eye examinations during this period. Thus, in summary, although these systems were successful in reducing the number of patients requiring face‐to‐face appointments during the COVID‐19 pandemic, it is unclear whether they have all remained in place long term.

### Comparing outcomes across interventions

**FIGURE 3:**
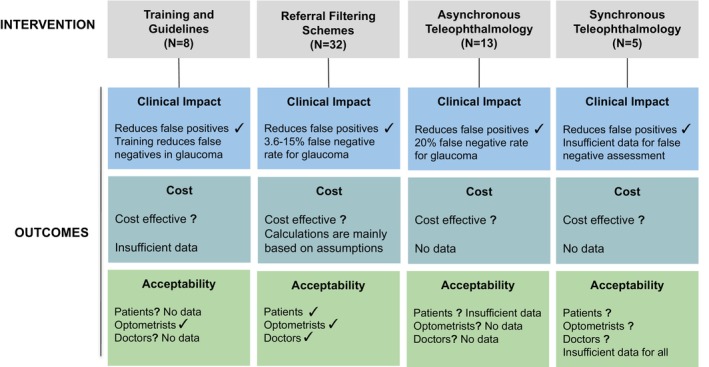
A summary of evidence in support of three outcome measures in relation to four types of intervention. Where the evidence supports the clinical outcome, a ‘✓’ is displayed. Where the outcomes are not fully supported or evidence is lacking, a ‘?’ is displayed. For outcomes which are not fully supported, the reason why this was decided is stated.

Based on the studies reviewed, we summarised the evidence presented in relation to three main outcomes used as measures of effectiveness: clinical impact, cost and acceptability. Figure 3 summarises these outcomes in relation to each intervention.

## DISCUSSION

In this section, we consider the impact of the different interventions on the three main stakeholder groups involved:

1. Patients
2. The Hospital Eye Service
3. Community optometrists.

### Impact on patients

We must consider the effect of new interventions on patients' safety and experiences. Although there was sufficient evidence to support a positive patient experience with relation to referral filtering schemes, there was insufficient evidence in relation to teleophthalmology interventions. Only one study of asynchronous interventions reported patient satisfaction outcomes using a binary measure^4^ and detailed opinions into which aspects of the service patients liked/disliked were lacking. Previous enquiries have investigated patient satisfaction with ophthalmology virtual clinics in more depth, mainly for follow‐up patients. Although findings have been generally positive, such as surveys reporting a similar mean satisfaction score compared to a standard clinic,^75^ there may be concerns around the lack of contact with a clinician, with some patients feeling that they would like a dialogue with a healthcare professional during each appointment.^76^

One potential positive impact of all the interventions explored is the reduced waiting time between patients being referred and their review. One study reported that in 96% of referrals, a HES specialist had virtually reviewed the referral and provided a working diagnosis/plan within the next calendar day.^63^ Reducing the number of false‐positive referrals seen face to face in the HES would also reduce waiting times for patients with ocular disease requiring hospital assessment and treatment. Patient care and treatment can be time critical. For example, it has been reported that for patients with wet age‐related macular degeneration, a delay in treatment of over 4 weeks can cause a loss of three lines in logMAR visual acuity.^77^ However, where referral is deemed necessary for patients, it could be argued that schemes such as enhanced referral, where an extra step is added to the pathway, may cause delay in accessing the HES and required treatment. Only low‐risk patients are therefore deemed suitable for these pathways.

The last significant patient factor to consider is the potential for false‐negative cases. These represent patients with referable ocular conditions requiring HES attention and/or treatment who are erroneously not referred to the HES. In the reviewed studies assessing referral filtering schemes, the false‐negative rate was up to 15%,^44^ while for asynchronous patient review it was 20%,^70^ which represents a relatively high percentage of discharged patients who were considered as requiring HES review in two studies. It should be noted that a comment published in Eye in 2022 (which did not meet our inclusion criteria for review) reported a CUES scheme false‐negative rate of just 0.23% for moderate‐to‐high risk of sight loss cases, which the authors described as ‘reassuringly low’.^78^ Combining more than one approach, such as referral filtering schemes with HES virtual review of discharged patients may increase clinical safety,^44^ but this would add another element of cost and resources where with adequate training, optometrists can perform safely, as demonstrated by the Manchester glaucoma enhanced referral scheme.^35^

### Impact on the hospital eye service

Despite there being a range of values for appointments avoided by different interventions, and some interventions only being appropriate for low‐risk referrals, the evidence suggests that all four classes of interventions can have a positive impact.

When assessing the effectiveness of the different interventions, we must also consider how the allocated work time of HES specialists may be impacted. For example, if clinicians are involved in a new pathway which includes synchronous or asynchronous review of patients using teleophthalmology, this must be an efficient use of their working hours. For asynchronous review of new referrals, there is a strong argument that this is an efficient use of time as patients can be triaged virtually in far less time than they would be if they were seen face to face in the clinic.^7^ One study reported that when using a cloud‐based referral system for suspected retinal disease, the mean review time for referral refinement was just 3.0 min in total.^7^ This is significantly shorter than a patient encounter in a face‐to‐face clinic and means that more patients could be reviewed by a HES clinician in the same period if seen virtually. In comparison, synchronous teleophthalmology requires specialists to virtually assess patients in real time, which is less time efficient.

### Impact on community optometrists

We must also consider the positive and negative effects that discussed interventions may have on optometrists and/or optometry practices. One positive impact of implementing some of these schemes is the potential for improved interaction between primary and secondary care. When using a typical referral pathway, after a patient is seen in the HES, a clinic letter is written by the healthcare professional which summarises the appointment findings but is usually addressed to the general medical practitioner only. Early studies found that referral reply rate to optometrists, either through direct reply or by copying in, varied from 13% to 16%^79^, ^80^ due to the general medical practitioner not always including the optometrists' contact details on the GOS referral, general medical practitioners do not see their role as one that passes on information to the referring optometrist and that optometrists are transient care providers.^81^ Feedback as part of new pathways such as direct referrals using virtual pathways could not only keep optometrists up to date with outcomes of patients for if/when they see them again in practice but would also act as a learning aid for when they encounter similar cases in the future, enabling them to make better management decisions.

New referral pathways/schemes must also be a beneficial and cost‐effective use of optometrists' time in practice. For the implementation of new direct pathways such as asynchronous, cloud‐based referral platforms, the systems must be intuitive for optometrists to easily refer patients in a time‐efficient manner. Similarly, for optometry practices to be willing to take part in referral filtering schemes, the clinical time allocated to seeing these patients must be cost‐effective for practices through sufficient remuneration from local or national funding. In England, the limitation of the GOS contract is one of the main issues with community eye care.^82^ Unlike the GOS contract in Scotland, there is no additional funding for supplementary tests and additional test time, which are essential for referral filtering. Local funding of such schemes presents an issue with their sustainability and creates differences in local guidelines between regions. Even with allocated funding, some practices may choose not to sign up to deliver a service such as MECS or to offer limited appointments, as the cost of the appointment may not be fully subsidised. Additionally, there is a potential loss of income when the purpose of the appointment is focussed on an ocular health concern. This poses a problem for optometric primary care which uses a cross‐subsidisation business model (i.e., using the sale of optical products to subsidise income lost from eye examinations).

### Missing information

There were two main features which were lacking in the body of reviewed literature. First, this review was intended to be a mixed‐methods review, whereby a broad range of literature of both quantitative and qualitative methods were included. However, although some included studies used qualitative methods, the vast majority were studies using quantitative measures. This meant that we were unable to gain qualitative insight into some of the interventions, and relied on speculation to determine explanations for the quantitative findings.

Second, we must also consider the potential use of AI to improve the accuracy of referrals entering the HES. A great deal of research is currently focussing on AI for aiding the diagnosis and management of ophthalmic conditions.^83^, ^84^, ^85^, ^86^ AI systems specifically for diabetic retinopathy screening in primary care are already being implemented and piloted in real‐world settings. A small number of non‐UK studies have reported on safe systems^87^, ^88^, ^89^ with a positive impact of increasing attendance when used as a point‐of‐care device,^88^ as well as potentially reducing the burden on current screening services.^87^ Although optometrists have expressed positive attitudes towards the future use of AI in primary care as a diagnostic tool for retinal disease,^90^ we found no investigations that have implemented or piloted AI specifically for the diagnosis/management of patients referred from primary care optometry to the HES. Protocols have been published which describe a pilot study^91^, ^92^ currently taking place in London using a cluster randomised trial to evaluate a teleophthalmology referral pathway for retinal disease. This includes assessment of the accuracy of an AI diagnostic support system for automated diagnosis and referral recommendation. However, results from this clinical trial are yet to be published.

### Limitations

We identified two main limitations to this review, which were based on our search strategy and inclusion/exclusion criteria. First, we specifically excluded studies that focussed on diabetic retinopathy assessment and screening. We made this decision as pathways for diabetic retinopathy referrals, certainly in the UK, do not follow the typical referral route from primary to secondary care. Patients with diabetes are usually seen within a screening service which runs parallel to standard pathways from primary care optometrists, so assessing interventions to this pathway would not fit in with our study focus. However, we acknowledge that over recent years, advances have been made in the use of AI technology for diabetic retinopathy screening and grading, and that we excluded four studies^86^, ^87^, ^88^, ^89^ specifically focussing on real‐world AI implementation which was lacking in the reviewed literature. Furthermore, we excluded studies not published in English, which could have limited diversity in relation to their country of origin.

Second, we used broad search and inclusion criteria in relation to study focus and design and completed no formal quality assessment of the included studies. Although this highlights a strength of our study, in that it allowed us to gain a broad overview of interventions which included both a quantitative and qualitative perspective, while considering a range of success factors, it meant that directly comparing studies was difficult and that a statistical approach was not appropriate.

### Clinical importance and conclusions

Overall, our review highlights that the implementation of a successful intervention for reducing false‐positive referrals is more complex than a ‘one‐size‐fits‐all’ approach. First, certain interventions are more established for specific eye conditions. Referral filtering schemes, for example, appear to run well for conditions such as glaucoma and cataract, but we found no evidence for similar schemes for routine referrals of suspected retinal conditions. This is perhaps due to referrals for suspected retinal disease being less frequent and more diverse, making it difficult to implement a structured refinement scheme. In contrast, using asynchronous review of clinical information by ophthalmologists is useful for the quick triage of suspected retinal conditions, but would not be appropriate for cataract referrals where a conversation around symptoms and willingness to undergo surgery must take place.

The effectiveness of each type of intervention also varies based on what outcome is being considered as a measure of success and which stakeholder is the focus. From the studies examined in this review, there was sufficient evidence to support the positive clinical impact of all interventions discussed, in reducing the false‐positive referrals being seen face to face within the HES, but evidence around cost‐effectiveness of all interventions is either insufficient or conflicting. Furthermore, more studies are required to explore stakeholder opinions around these interventions, and we acknowledge that there is less of a drive to publish negative stakeholder views when schemes produce clear benefits for easing the strain on the HES.

In order to maximise the safety of these interventions, it may be useful to combine more than one approach, such as referral filtering schemes with virtual review of discharged patients or for some community schemes such as MECS to be operated only by those with extra training in independent prescribing. Of course, this would require additional costs and resources. Although our literature search found no assessment of implemented AI systems for our specific focus, the increasing availability of AI systems means that there is potential for AI to play a role in clinical decision support systems within referral pathways from primary care in the future.

## AUTHOR CONTRIBUTIONS

**Josie Carmichael:** Conceptualization (lead); data curation (lead); formal analysis (lead); writing – original draft (lead). **Sarah Abdi:** Data curation (supporting); writing – review and editing (equal). **Konstantinos Balaskas:** Funding acquisition (equal); supervision (supporting); writing – review and editing (equal). **Enrico Costanza:** Supervision (supporting); writing – review and editing (equal). **Ann Blandford:** Conceptualization (supporting); funding acquisition (equal); supervision (lead); writing – review and editing (equal).

## CONFLICT OF INTEREST STATEMENT

The authors have no conflicts of interest to declare.

## Supporting information


Data S1.
