# Supplementary material for: The effectiveness of interventions for optometric referrals into the hospital eye service: A review
Source: Ophthalmic Physiol Opt. 2023 Aug 25;43(6):1510–23. doi: 10.1111/opo.13219 (PMC10947293; doi:10.1111/opo.13219)
Supplement: Supplementary file 1 — Supplementary file (DOCX 66.3 KB) [file 44402_2023_4306022_MOESM1_ESM.docx]

| **Author(s)** | **Year** | **Location** | **Study Period** | **Study Design** | **Aim** | **Condition(s)** | **Intervention** | **Main Results** |
| --- | --- | --- | --- | --- | --- | --- | --- | --- |
| **Cottrell et al.** | 2022 | UK (Wales) | April 2020-June 2020 | Online Survey | To describe optometrists' independent prescribing (IP) practices during the COVID-19 pandemic in Wales. | All ocular conditions | Independent prescribing training and qualification | 81 practices conducted 22,434 interactions. 80.26% were self-referred. **Prescriptions:** 1435 medications were prescribed, of which 1332 (92.82%) were topical. 1136 (79.16%) of prescriptions were issued in health boards with IPOS services, 288 (20.07%) in health boards with prescribers but no IPOS and 11 (0.77%) in areas with no prescribers. **Patient outcomes**: 2071 (9.23%) appointments ended in a referral to ophthalmology, 1300 (5.79%) to GPs, 1251 (5.58%) to pharmacies and 307(1.37%) to other professionals.  Health boards with IPOS had fewest total and urgent referrals to ophthalmology. Health boards with no prescribing saw the highest proportion of referrals for urgent ophthalmology assessment.  Significant association between the prescribing group and referral rates for urgent ophthalmology referrals (p < 0.001), and referrals to GP (p= 0.001), with a higher proportion of referrals made in non IPOS areas. There was no association between routine referral to ophthalmology and prescribing group (p = 0.16) |
| **El-Abiary et al.** | 2021 | UK (Scotland) | 2010-March 2019 | Quantitative retrospective analysis of optometrists and referrals | To identify the distribution of IP optometrists across Scotland and assess the impact of IP on referral rates into HES. | All ocular conditions | Independent prescribing training and qualification | 278/1189 (23.4%) community optometrists in Scotland hold IP qualification. In 2019, there was no association between the quantity of IP optometrists and the referral rate to HES (Pearson correlation coefficient r = +0.53, p = 0.052). |
| **Sii et al.** | 2019 | UK(Scotland) | October–November 2014 (group 1)  September–October 2016 (group 2) | Retrospective analysis of 312 (group 1) and 325 (group 2) patients from two areas seen in the HES. | To assess the impact of Scottish Intercollegiate Guidelines Network (SIGN) 144 on quality of referrals from community optometrist | Glaucoma | SIGN 144 Guidelines | **First visit discharge:** Patients referred post-SIGN were less likely to be discharged on their first visit (p=0.004). The overall FVDR declined from 29.2% to 19.4% following the introduction of SIGN guidelines. FVDR pre-SIGN were mainly referrals for high IOP (40%), abnormal optic disc (25%) and abnormal visual field (24%). Post-SIGN guideline implementation, first visit discharges were mainly referrals for abnormal optic disc (31%), abnormal visual field (21%) or both (19%). **Compliance with guidelines:** 86% of referrals post-SIGN implementation were found to be compliant under one or more categories for referral.  There was an increase from 36.5% to 53.9% in referrals with repeated IOP readings. There was an increase from 58.8% to 79.6% in IOP using contact tonometry.  Visual field assessment repeating increased from 31.7% to 42.8%.  Cup: disc ratio measurement increased from 58.8% to 83.6%, and attachment of disc images increased from 7.7% to 36.8% |
| **El-Assal et al.** | 2015 | UK | June 2000–May 2006 (Group A) January 2007–December 2012 (Group B) | Quantitative retrospective audit of new HES glaucoma patient records. Group A (n=835)  and Group B (n=737) | To evaluate accuracy and outcome of community optometry referrals after implementation of the new 2006 GOS contract, the 2008 Eyecare Integration Programme pilot and the 2009 NICE guidelines. | Glaucoma | New GOS contract (2006), the Eyecare Integration Programme pilot (2008) and the NICE guidelines (2009) | Waiting times reduced from 12.3(Group A) to 9.4 weeks (Group B). Significantly more patients kept first appointment (p = 0.0002) in group B.  At the first hospital appointment 633 eyes (37.6 %) were found to be normal in group A compared to 380 eyes (24.1 %) in group B.  There were significantly fewer normal patients (p < 0,0001), more glaucoma suspects (p < 0.0001), more open angle glaucoma patients (p = 0.0006) and fewer other conditions (p = 0.0024) in group B, compared to group A. |
| **Needle et al.** | 2008 | UK | July-August 2006 (6 weeks) | Online survey from 1269 optometrists (including multiple choice and free-text responses) | To investigate optometrists' clinical practice and to elicit their views on the independent prescribing role | All ocular conditions | Independent prescribing training and qualification | Most optometrists felt that, with training, they should be able to prescribe classes of ophthalmic drug (range 58–84%) except for corticosteroids (44.1%).  8% of respondents were currently training for an extended prescribing role. Hospital optometrists expressed the most interest in extended prescribing and were more likely to be either in training (17%) or actively considering training (38%) for supplementary prescribing (p< 0.001 and p= 0.004, respectively).  The 29% of respondents who were actively considering training were likely to be more recently qualified (p< 0.001) 9% said that they had no intention of undergoing further training for prescribing.  The most significant barriers to undertaking the training were remuneration (70%), fear of litigation (58%) and the lack of time (64%) or cost of training (61%).  Respondents expressed annoyance at the length of placements (% not given). |
| **Dahlmann-Noor et al.** | 2007 | UK | 3-month period in 2003 and 7-week period in 2006 | Quantitative prospective analysis of 159 referrals and quantitative retrospective analysis of 185 case notes. | To evaluate the quality of the West Suffolk Direct Referral Scheme | All ocular conditions | Six-monthly training sessions and regular feedback via letter about consultation outcomes | 99% referrals were appropriate.  Diagnostic competence was 87% and improved with tighter communication between HES and optometrists.  Agreement remained unchanged for urgency (75%) and decreased for choice of subspecialty clinic from 88% to 74% (due to a larger number of cases being channelled into direct referral clinics for ease of access, despite optometrist’s requests for subspecialty appointments) |
| **Patel et al.** | 2006 | UK | June 2002-May 2003 | Quantitative retrospective analysis of 376 referrals | To determine if the effect of training intervention on the accuracy of glaucoma referrals and to see if increased numbers of glaucoma cases detected was achieved. | Glaucoma | Training in optic disc assessment and referral criteria every 4 months | 58% (376/238) increase in the number of referrals. Positive outcome in 171/376 of referrals (PPV =0.45 (95% CI 0.41–0.51)).  From the intervention group 93/183 resulted in a positive referral (PPV = 0.51 (95% CI 0.44–0.58))  From the control group 35/86 were positive referrals (PPV = 0.41 (95% CI 0.31–0.51)).  From the non-randomised group 22/59 resulted in positive referral (PPV 0.37 (95% CI 0.26–0.50)). |
| **Theodossiades et al.** | 2004 | UK | June 2000-January 2001 | Randomised control trial. 119 referrals control arm and 210 intervention arms. Mixed methods | To test an intervention aimed at  improving optometrist case-finding | Glaucoma | Training in optic disc assessment and referral criteria | **Outcomes:** 102/210 of all referrals from the intervention group resulted in a positive outcome (PPV 0.49). 55/119 of all assessed referrals from the control group resulted in a positive outcome (PPV 0.46). **Interviews:** All 13 optometrists reported adopting a more comprehensive analysis of the optic discs since the training. The majority of the 13 reported that they were happy with the content of the training. |

**Supplementary Table 1**: Summary of studies focusing on training and/or guidelines.

| **Author(s)** | **Year** | **Location** | **Study Period** | **Scheme** | **Study Design** | **Aim** | **Results** |
| --- | --- | --- | --- | --- | --- | --- | --- |
| Wang et al. | 2021 | Australia | July 2016 and June 2019 | Non-urgent ocular pathology | Retrospective analysis of 755 patients seen in the CFEH | To evaluate the CFEH integrated eye-care model in the identification of chronic eye diseases within the community. | **Diagnosis**: Approximately half of eye condition-specific appointments at CFEH were glaucoma-related (48.8%) with the majority of remaining appointments consisting of retinal assessments. 77.4% of assessments resulted in the diagnosis of an eye condition or identification of patients at a moderate or high risk of developing eye conditions.  2.6% of patients referred had no evidence of ocular pathology. 15.5% of patients were found to have incidental or concomitant pathology with almost half of this cohort requiring same day intervention. **Management**: 200 (26.5%) were discharged, 432 (57.2%) were recommended monitoring at CFEH and 123 (16.3%) were referred onward to ophthalmology. While most referrals were non-urgent (68.7%), 8.0% required same day referral and 19.6% had a recommended referral time frame within 4 weeks. Most patients requiring onward referral to ophthalmology had their clinical findings confirmed by an ophthalmologist (93.5%) while 1.1% of patients were discharged. |
| **Kanabar et al.** | 2021 | UK | Primary care: 1st June-31st July 2020  Secondary care: 17th June-11th August 2020 | Urgent cases | Quantitative retrospective and prospective analysis  of referrals. | To evaluate the COVID-19 urgent  eye care service (CUES) for primary and secondary care activity. | 91.1-91.7% were initially deemed eligible for a telemedicine appointment. 53.3-55.6% were given face-to-face appointments following a telemedicine appointment. 13.0-14.3% of cases were eventually provisionally referred to secondary care HES. Of the 101 provisional referrals to MREH from CUES received, 69 (68.3%) were accepted  Of the 61 accepted referrals graded by the hospital clinicians, 39 (63.9%) were categorised as either being in ‘agreement’ or ‘partial agreement’. Of the 32 rejected referrals, 25 (78.1%) were rejected due to the condition not being deemed an emergency 420 telephone calls were recorded and signposted to either CUES, the MREH EED, or local hospitals/optometrist practices. In 56.0% (235 phone calls) the patient was advised to attend MREH EED and in 32.4% (136 phone calls) the patient was advised to see a CUES optometrist in the community. |
| Huang et al. | 2020 | Australia | March 2015-June 2018 | Glaucoma | Quantitative retrospective, analysis of 252 glaucoma referrals | To examine the impact of referral source (community optometrists vs RR) on patient glaucoma management | A significantly higher proportion of patients were confirmed with a glaucoma diagnosis following referral refinement (43.8%) compared to community referrals (27.0 %, p = 0.008) PPV for referral refinement was 51% (90/178) and 34% (25/74) for community referrals.  False positive referral rates were 4% for referral refinement (8/178) and 26% (19/74) for community referrals.  Patients having undergone referral refinement were more likely to result in treatment initiation compared to those referred directly from a community optometrist (p = 0.016) |
| Phu et al. | 2020 | Australia | Pre-suite August 2017-February 2018 Post-suite/angle suite March 2018-August 2018 | Glaucoma (Angle suite) | Quantitative retrospective analysis of patients seen pre (n=383) and post (n=425) introduction of a referral pathway for anterior chamber angle assessment (Angle Suite). Patients seen via the angle suite were also analysis (n=77). | To evaluate a newly developed referral and collaborative care pathway specifically for patients with angle closure spectrum disease | **Waiting times:** Angle Suite patients had a significantly shorter time to appointment compared to both Pre Suite and Post Suite groups (p< 0.0001). Post Suites had a shorter time to appointment to Pre Suites (p = 0.0002). The Post Suite cohort had an approximately one-third reduction in angle closure diagnosis compared to the Pre Suite cohort (6.6% vs 4.0%, p = 0.1189) 13.6% of patients had a stage of angle closure disease that required prompt intervention in the Pre Suite and 9.3% in the Angle Suite groups. No patient in the Post Suite group required urgent referral. The true negative rate (open angles mentioned in the letter and open angles found) was 100% (28/28) for the Pre Suite and 92.9% (52/56) for the Post Suite plus Angle Suite. The true positive rate (narrow angles mentioned in the letter and angle closure glaucoma spectrum disease found) was 73.1% (19/26) for the Pre Suite and 70.1% for the Post Suite plus Angle Suite (54/77).  The proportion of cases diagnosed with angle closure spectrum disease in the Pre and Post period where the angle was not described in the referral letter were 37.5% (9/24) and 75.0% (12/16), respectively. |
| Ford et l. | 2019 | Australia | Standard pathway: October 2014-April 2017 C-EYE-C pathway January 2017–October 2017 | Glaucoma | Retrospective clinical and financial audit o 182 standard pathway referrals and 321 C-EYE-C referrals | To determine whether C-EYE-C improves  access to care and better utilises resources, compared to hospital-based care. | **Waiting times:** The C-EYE-C model demonstrated a significantly shorter median wait-time from referral to first appointment of 89 days compared to 386 days for standard care (p < 0.001.)  **Outcomes:** The total proportions of patients diagnosed as a glaucoma suspect, with definitive glaucoma, or glaucoma with additional ocular pathology was 76% for the standard pathway and 90.9% for the C-EYE-C. Over half of the patients in both standard pathway and C-EYE-C (57.6% Vs 56.5%) required routine follow-up (>3 months) **Appointments avoided:** There were 148 hospital outpatient appointments avoided by patients that attended the C-EYE-C clinic for the first encounter. Assuming that the outpatient clinic has 14 glaucoma appointments available each week for new patients, then 10.6 weeks of appointments were saved by assessing patients off-site at C-EYE-C. **Diagnostic agreement**: Absolute agreement between C-EYE-C and virtual ophthalmologist was 68% and a 95% weighted agreement (k = 0.69). For patient management decisions the absolute agreement was 79%, with a weighted agreement of 95% (k = 0.66). For cases where the optometrist’s recommendation was changed, 7.6% required more urgent care, and 13% less. Numbers of patients discharged did not change. |
| Gunn et al. | 2019 | UK | October 2014-August 2016 | Glaucoma | Prospective, quantitative analysis of 1404 patients evaluated in GERS | This evaluates the clinical effectiveness of the Manchester Glaucoma Enhanced Referral Scheme (GERS). | **False positives:** The FP rate (patients discharged at first visit) was 15.5% (44/283) 54.1% (153/283(were monitored in the HES without treatment, 27.6% (78/283) were monitored with treatment, 3.2% (9/283) required further investigation. **False negatives:** 89.3% (117/131) seen by the GERS and not referred were confirmed as not requiring hospital follow-up.10.7% (14/131) required follow-up, including 5 (3.8%) offered treatment. Only one patient (0.8%) in this sample met the GERS referral criteria and was not referred (true FN) |
| Konstantakopoulou et al. | 2018 | UK | September 2013-August 2014 | MECS | Quantitative prospective analysis of 2123 patients evaluated in the MECS | To monitor the activity and evaluate the clinical  safety of a MECS | 75.1% (1595/2123) of MECS patients remained within community optometric practice; 64.0% (n=1359) were diagnosed with pathology and managed in the community. 11.1% (236/2123) were found to have no pathology and discharged. 5.7% (122/2123) were referred to their GP and 18.9% (400/2123) were referred to the HES. 49.1% were routine, 22.6% urgent and 28.3% emergency For a sample MECS assessments reviewed by the research team, 5.5% (12/220) were rated as inappropriate. 3(1.36%) patients rated as inappropriate management could have come to harm by the optometrists’ management  89.2% were judged to have been appropriately referred and 78.2% were referred with appropriate urgency. |
| Ly et al. | 2017 | Australia | 1st July 2013-30th June 2016 | Pigmented lesions | Quantitative retrospective review of 182 patients referred to an intermediate-tier clinic (CFEH). | To describe the referral patterns of pigmented lesions to an optometry led intermediate-tier collaborative clinic. | **Diagnosis**: Choroidal naevus was the suspected diagnosis in 58% (105/182) and CFEH diagnosis in 59% (107/182). The number of cases without a specific diagnosis was reduced by approximately two-thirds (29% to 10%) after assessment at the CFEH.  **Management:** The CFEH report most frequently recommended recall for CFEH review (53%, 96/182), followed by discharge (35%, 64/182), or referral to an ophthalmologist (12%, 22/182). |
| Ly et al. | 2016 | Australia | 1st July 2013-30th June 2014 | non-urgent macular disease | Quantitative retrospective review of 291 patients referred to an intermediate-tier clinic. | To appraise the optometric referral patterns of  patients with suspected macular disease to an  intermediate-tier optometric imaging clinic | **Diagnosis:** The most common diagnoses suspected by primary care optometrists was non-neovascular AMD (75, 26%), CSCR (22, 8%) and ERM (8, 6%). 3 cases were referred to confirm that the macula was normal.  AMD was the most common diagnosis (93, 32%) after assessment at CFEH, followed by other (54, 19%), ERM (22, 8%), normal aging changes (21, 7%), no apparent defect (NAD; 22, 8%) and CSCR (13, 4%). The number of cases without a diagnosis was halved (reduced from 47% to 23%). Cases with NAD rose from 1% to 8%.  121/291 (42%) referrals stipulated a suspected diagnosis that was confirmed after evaluation at CFEH **Management:** 244/291 (84%) patients were recommended ongoing optometric care: with the referring optometrist (57/291, 20%) or through recall to CFEH (187/291, 64%). Referral to an ophthalmologist was recommended in 47/291 (16%). |
| Konstantakopoulou et al. | 2016 | UK | September 2013-August 2014 | MECS | Retrospective, quantitative analysis of 2123 MECS appointments. Qualitative analysis of patient satisfaction questionnaires. | To evaluate the clinical effectiveness, impact  on hospital attendances and patient satisfaction  with MECS | **Outcomes:** 64.1% were managed by optometrists and 11.2% were discharged with no ocular pathology. 18.9% of patients were referred to the HES, of which 49.1% were referred routinely, 22.6% urgently and 28.3% emergency. Based on a consensus panel assessment 95% (208/220) of a sample were appropriately managed.  **Management agreement:** 89.2% were judged as referred appropriately and 78.2% were referred with appropriate urgency. For inappropriate referrals, in over 90% these were referred with greater urgency than required.  First attendances to the HES referred by GPs dropped by 26.8% and follow-up appointments fell by 12.9% in the areas operating the MECS scheme compared to the comparison area. |
| McAlinden et al. | 2016 | UK | February 2012 | WEHE and PEARS | Quantitative prospective analysis of 2302 patients seen in the WEHE or PEARS scheme. | To assess the demographics of patients accessing WEHE/PEARS, referral patterns and clinical management. | **Outcomes:**27.8% (640/2302) required no further action and were discharged. 43.3% (997/2302) required monitoring by their optometrist or ophthalmic medical practitioner, 15.9% (367/2302) required referral to the HES, 7.3% (168/2302) required referral to the GP. The GP was informed in 53.2% (1223/2302) |
| **El-Assal et al.** | 2015 | UK | June 2000–May 2006 (Group A) January 2007–December 2012 (Group B) | New GOS contract (2006), the Eyecare Integration Programme pilot (2008) and the NICE guidelines (2009) | Quantitative retrospective audit of new HES glaucoma patient records. Group A (n=835)  and Group B (n=737) | To evaluate accuracy and outcome of community optometry referrals after implementation of the new 2006 GOS contract, the 2008 Eyecare Integration Programme pilot and the 2009 NICE guidelines. | Waiting times reduced from 12.3(Group A) to 9.4 weeks (Group B). Significantly more patients kept first appointment (p = 0.0002) in group B.  At the first hospital appointment 633 eyes (37.6 %) were found to be normal in group A compared to 380 eyes (24.1 %) in group B.  There were significantly fewer normal patients (p < 0,0001), more glaucoma suspects (p < 0.0001), more open angle glaucoma patients (p = 0.0006) and fewer other conditions (p = 0.0024) in group B, compared to group A. |
| Roberts et al. | 2015 | UK | February 2005-February 2009 | Glaucoma | Quantitative retrospective analysis of 1639 patients seen in the refinement scheme. | To report on results of a glaucoma shared-care scheme based in Peterborough, UK. | **Waiting times**: The median waiting time between referral and SOG assessment was 0 days and the median time between SOG assessment and ophthalmologist evaluation was 12 days, **Diagnosis:** 18.3% of patients were diagnosed with glaucoma, and in 5.8% no pathology was found. Most patients (65.4%) were diagnosed as glaucoma suspects, had OHT or risk factors for glaucoma. A minority were found to be at risk of angle closure or had other pathology (5.6 and 1.5%, respectively).  **Diagnostic agreement**: Level 2 SOGs had 64.6% agreement with a consultant, 23.2% non-significant disagreement, 5.6% disagreement. Level 1 SOGs had 47.5% agreement, 28.4% non-significant disagreement, 15.3% disagreement. **Outcome**: Level 2 SOGs had a 69.5% agreement, falling to 49.1% in the Level 1 SOGs. Non-significant disagreement was 18.7 and 21.0% and disagreement was 10.4% and 28.6% in for Level 2 and Level 1 SOGs respectively. **Sensitivity/specificity**: Level 2 SOG’s had a sensitivity of 61.0% and a specificity of 75.2%. The sensitivity and specificity of Level 1 SOGs was 53.8% and 64.8%. |
| Keenan et al. | 2015 | UK | 1st April 2010- 31st March 2013 | Glaucoma | Retrospective, quantitative analysis of 1733 patients seen as part of the refinement scheme. | To describe outcome data from the Cambridge  community Optometry Glaucoma Scheme (COGS) | Following assessment, 46.6% (n= 807) patients were discharged by an OSI.  **Management agreement:** Consultant ophthalmologist agreement with OSI management decisions was 91.5%. Following virtual review of patient data, a further 5.7% (n= 99) patients were discharged. Virtual review resulted in 3.6% of all patients (n= 62) who had been discharged following community OSI assessment being recalled to the HES. Following further assessment in consultant-led clinic, 11 of the recalled patients were discharged at first visit. Of the 111 OSI referrals for an occludable anterior chamber angle, the consultant ophthalmologist found 43 (38.7%) patients to have narrow angles on gonioscopy. |
| Ratnarajan et al. | 2015 | UK | __ | Glaucoma | Retrospective quantitative assessment 120 seen in a glaucoma referral refinement scheme. | To establish the safety of the CHANGES glaucoma referral refinement scheme (GRRS). | 46/120 (38%) of patients seen in the glaucoma refinement scheme were discharged and 34/46 (74%) of the agreed to attend a HES review by the glaucoma consultant.  **Management agreement:** The glaucoma consultant found all 34 patients to have GAT IOP measurement below the JCG threshold for discharge. 5/34 (15%) were found by the consultant to have a suspicious optic nerve following slit lamp biomicroscopy, were classified as ‘glaucoma suspect’ and offered a follow-up appointment. This translates to a ‘missed glaucoma rate’ of 0% and a false negative rate of 15% for the OSI. This rate is not for the CHANGES scheme as a hospital optometrist virtually reviews the digital images of all optic discs of patients discharged. |
| jan et al. | 2013 | UK | March-April 2011 | Glaucoma | Retrospective, quantitative, multisite analysis of 271 patients (from Huntingdon, Manchester, Gloucestershire and Nottingham). | To compare glaucoma referral refinement schemes (GRRS) in the UK during a time period of considerable change in national policy and guidance. | For OSIs, first visit discharge rate (FVDR) 17.2%  For non-OSIs FVDR was 43.9%  The largest source of first-visit discharges for both non-OSIs and OSIs was for IOP-only related referrals (83.5% and 55% respectively)" |
| Ratnarajan et al. | 2013 | UK | August 2006-June 2011 | Glaucoma (referral refinement with  shared care) | Quantitative retrospective audit of 912 glaucoma referrals | To assess the impact of referral refinement criteria on the number of patients referred to, and first-visit discharges from, the HES | **Raised IOP:** 429 referrals from community optometrists were due to raised IOP (22–28 mmHg), of which 34% were discharged by the OSI. 38 referrals were for IOP asymmetry >5 mmHg of which 45% were discharged by the OSI. **Abnormal optic disc**: 207 referrals from community optometrists were for an abnormal optic disc alone, of which 37.7% were discharged by the OSI. **Abnormal VF**: 84 referrals from community optometrists were for an abnormal VF alone, of which 51% were discharged by the OSI. **JCG guidance:** 51/70 (73%) patients who were aged between 65–80 and 6/10 (60%) who were aged over 80 and had been referred by OSIs on the basis of raised IOP only would have satisfied the JCG criteria for non-referral. |
| Parkins and Edgar | 2011 | UK | April 2007-April 2008 | Glaucoma | Quantitative retrospective analysis of glaucoma referrals seen via one of referral schemes (209 from repeat measures and 218 for referral refinement). | To compare the clinical and financial effectiveness of two optometric-led enhanced glaucoma referral schemes | **Repeat Measures:** 50 (24%) patients were referred on to the HES. In 57 (44.5%) of the 128 cases where raised IOP by NCT was found repeated measurement by Goldmann/Perkins applanation tonometry resulted in lower readings of 21 mmHg or less, or less than a 5-mmHg difference between the two eyes. **Referral Refinement**: After reviewing initial referrals, 111 patients (51%) were referred direct to the HES and 107 to the refinement scheme. The scheme referred 12/107 (11%) patients for investigation for suspect glaucoma. They discharged 76 patients (71%) and booked 15 for further refinement. Ten of these patients were subsequently discharged. |
| Devarajan et al. | 2011 | UK | 4-year period | Glaucoma | Retrospective analysis of 100 patients referred to the HES via, and 100 patients discharged from a refinement scheme. | To describe a community glaucoma refinement scheme. | **Outcomes:** 83% of all referrals from the refinement scheme were either diagnosed immediately with glaucoma or retained in the clinic for follow-up investigation. Of the 14 ‘normals’, only 5 were immediately discharged, Visual-field abnormalities were diagnosed in 51% on referral, compared to 43% in the HES.  **False negative rate**: All patients in the sample of discharged patients (n=100) were found to have followed the agreed protocols. Of the 98 virtually reviewed discharged patients, consultant ophthalmologists were in agreement with the referring optometrist 50% of the time, suggested overestimation of CDR for 35% of images, and underestimation for 15% (of which 2 showed changes that merited recall to the HES for investigation, but neither were started on treatment. This translates as a false-negative rate of 3-10%. |
| Syam et al. | 2010 | UK | February 2005-March 2007 | Glaucoma | Retrospective, quantitative analysis of 1184 glaucoma referrals and 72 patient satisfaction surveys. | To assess the role of specialist optometrists working in the community shared care for glaucoma patients. | **Waiting time**: Average waiting time from referral to SOG assessment was 36 days and between SOG assessment to HES evaluation was 15 days.  **Diagnostic agreement**: A significant disagreement between the appraisal and findings of the SOGs was observed in optic nerve morphology (11%), visual field (7%), diagnosis (12%), treatment (10%), and follow-up (17%) 68% of patients were followed up in the community. 32% of patients were referred to the HES. |
| Bourne et al. | 2010 | UK | 25th August 2006 -31st December 2007 | Glaucoma | Quantitative prospective assessment of 121 referrals triaged into and seen by a referral refinement scheme | To describe the design, activity, and quality of the referral refinement phase of a novel glaucoma shared-care scheme | The OSI discharged 35% 40/121 of patients seen. **Management agreement**: A consultant agreed (virtually) with the decision to discharge in 28/40 (70%). Compared to a consultant, OSI sensitivity for suspicious optic discs was 78%, specificity 61% and NPV 79%. OSI sensitivity for an IOP of >21 mmHg was 74%, specificity 85, and NPV 90%. OSI sensitivity for an occludable anterior chamber angle (Van Herick Vs gonioscopy) was 69%, specificity 88%, and NPV 94%. **Longitudinal:** When separating into two 8-month period to test for change over time, significantly fewer false positives were made by the OSI in the more recent 8-month period for IOP measurements only (p= 0.015). |
| **Ang et al.** | 2009 | UK | Pre-GOS June-November 2005 Post-GOS June-November 2006 | Glaucoma | Retrospective quantitative study of 183 referrals made during the first 6-month period and 120 referrals made during the second 6-month period. | To assess the quality of referrals from community optometrists in the Scotland to the HES before and after the implementation of the new General Ophthalmic Services (GOS) contract | **Patient outcomes:** The number of true-positive referrals after the new GOS contract 38/120 (31.7%) compared to before it was introduced 33/183 (18.3%) (p=0.006).  The proportion of patients discharged at the first visit was less post-GOS introduction 20/120(16.7%) compared to before it was introduced 79/183(43.2%) (p=0.004). **Quality of referrals:** post-GOS introduction, there was an improvement in the number of referrals with applanation IOPs (p=0.000), dilated fundal examination (p=0.000), and repeat VFs (p=0.004). Referrals with optic disc assessment and documentation of family history of glaucoma were lower (p= 0.017 and 0.050, respectively). Less than half (41.7%) fulfilled the new GOS (Scotland) contract requirements. The most common examination missing in the referral was applanation tonometry |
| Sheen et al. | 2009 | UK | April-December 2006 | PEARS and WEHE | Quantitative prospective analysis of 6432 patients and telephone interviews with a subset of 289 patients. | To derive an evidence, base for the efficacy of two optometric primary eye care services in Wales (PEARS and WEHE) | **Overall:** 66% (4243/6432) were managed in optometric practice without referral. 18% (1171/6432) were referred to the HES; and 16% (1018/6432) were referred to the GP, either for co-management (415; 41%) or for systemic investigation (603; 59%). **Patients referred to HES**: 75% were deemed to have been appropriately managed by the optometrist and 72% (284/392) correctly diagnosed. 73% (286/392) attended for at least two follow-up HES visits. Of the remaining 106, 85 (22%) were discharged at the first visit without treatment. |
| Henson et al. | 2003 | UK | _ | Glaucoma | A quantitative retrospective analysis of 194 patients who  had passed through the refinement scheme. | To describe a glaucoma referral refinement scheme and report the first year's results and its financial costs to the NHS. | **Outcomes:** 58% (112/194) of patients seen within the scheme were referred to the HES. |

**Supplementary Table 2**: Summary of studies focusing on the clinical impact of enhanced referral refinement schemes.

| **Author(s)** | **Year** | **Location** | **Study Period** | **Scheme** | **Study Design** | **Aim** | **Results** |
| --- | --- | --- | --- | --- | --- | --- | --- |
| Wang et al. | 2021 | Australia | July 2016 and June 2019 | Non-Urgent ocular pathology | Quantitative retrospective analysis of 755 patients seen in the CFEH | To evaluate the CFEH integrated eye-care model in the identification of chronic eye diseases within the community. | **Cost:** The average cost per patient assessment was 245 AUD. With an average rebate of 50.26 AUD from Medicare, the net cost of an eye disease assessment at CFEH is 195.50 AUD. There is no apparent cost reduction compared to the public hospital system. |
| Forbes et al. | 2019 | UK | April 2013-November 2016 | Glaucoma | Cost analysis of 2405 patient appointments | To examine the cost consequences of the Manchester Glaucoma Enhanced Referral Scheme (GERS) by considering the total costs of the scheme | Assuming 2.3 outpatient visits to the HES avoided per person: NHS cost saving of £6635 (approx. £2.76 per patient passing through the scheme). Assuming 1 HES outpatient visit was avoided per person, there was no cost saving and costs £101690 (approx. £42.28 per patient within the scheme) Patients need to have an average of 2.22 visits to the HES prior to discharge to make the GERS scheme cost neutral |
| Ford et l. | 2019 | Australia | Standard: October 2014-April 2017 C-EYE-C January 2017–October 2017 | Glaucoma | Retrospective clinical and financial audit of 182 standard pathway referrals and 321 C-EYE-C referrals | To determine whether C-EYE-C improves  access to care and better utilises resources. | **Cost Analysis:** The average cost per patient encounter was $171.00 for the hospital model, and $133.16 for C-EYE-C |
| Mason et al. | 2017 | UK | 2nd September 2013-30th August 2014 | MECS | Retrospective audit, with cost analysis of MECS scheme compared to a control area. Difference-in-difference comparison. | To examine how the introduction of MECS affected  the numbers of patients treated by the HES and the cost consequences | **Intervention area 1**: Total costs for HES and ITS activity were 2.5% higher in 2013–2014 (post-intervention) compared with 2011–2012(pre-intervention). **Intervention area 2**: Total costs for HES and ITS activity were 13.8% lower in 2013–2014 (post-intervention) compared with 2011–2012(pre-intervention) **Control area**: Total costs for HES and ITS activity were 3.1% higher in 2013–2014 (post-intervention) compared with 2011–2012(pre-intervention) |
| Ratnarajan et al. | 2013 | UK | August 2006-June 2011 | Glaucoma | Retrospective audit of 912 glaucoma referrals | To assess the impact of referral refinement criteria on the number of patients referred to, and first-visit discharges from, the HES | **Cost analysis:** The number of patients attending the HES was reduced by 15% in 2010. The cost saving of the CHANGES scheme was £16 258, which represents a 13% reduction compared to if all patients were seen directly by the HES. |
| Devarajan et al. | 2011 | UK | 4-year period | Glaucoma | Quantitative retrospective analysis of 100 referred to the HES via, and 100 discharged from a refinement scheme. | To describe a community glaucoma  refinement scheme. | **Cost Analysis:** The scheme resulted in a 53% reduction in the total number of referrals to HES with a cost saving of £117 per patient. |
| Parkins and Edgar | 2011 | UK | April 2007-April 2008 | Glaucoma | Quantitative retrospective analysis of all glaucoma referrals seen via one of two referral schemes (209 from repeat measures and 218 for referral refinement). | To compare the clinical and financial effectiveness of two optometric-led enhanced glaucoma referral schemes | **Repeat Measures:** The cost saving for the scheme was calculated as £17067 (62%) for the 209 patients, compared to if they were seen at the HES on first visit. **Referral Refinement**: The cost saving for the scheme was calculated as £1022 (3.5%) for the 218 patients, compared to if they were seen at the HES on first visit. |
| Sheen et al. | 2009 | UK | April-December 2006 | PEARS and WEHE | Prospective, quantitative analysis of 6432 patients and telephone interviews with a subset of 289 patients. | To derive an evidence, base for the efficacy of two  novel optometric primary eye care services in Wales | **Cost**: The net cost of the 6423 examinations over the 8- month period was approximately £77 000, or a cost of approximately £12 per PEARS or WEHE consultation. A cost model based upon a 50% referral to the HES with the remainder consulting the GP on two further occasions yields a cost of approximately £15 per PEARS or WEHE consultation |
| Henson et al. | 2003 | UK | _ | Glaucoma | A retrospective analysis of 194 patients who  had passed through the refinement scheme. | To describe a glaucoma referral refinement scheme and report the first years' results and its financial costs. | The cost saving works out to be approximately £17 per patient passing through the scheme. This is assuming that the training programme will have to be repeated every 3 years and that the scheme will continue to see 23 patients/month and 42% of these will not be referred to the HES. |

**Supplementary Table 3**: Summary of studies focusing on the cost-effectiveness of enhanced referral refinement schemes.

| **Author(s)** | **Year** | **Location** | **Study Period** | **Scheme** | **Study Design** | **Aim** | **Results** |
| --- | --- | --- | --- | --- | --- | --- | --- |
| Barrett and Loughman | 2018 | Ireland | _ | Glaucoma and MECS | Qualitative study using an anonymous  survey from 199 optometrists. | To explore optometrists’ attitudes towards  an enhanced scope of clinical practice | **Optometrists:** 4/199 participants (2.1%) indicated 'no interest in changing the scope of the traditional eye examination’, the remainder indicated varied levels of interest in expanding their scope of practice. 68% of respondents indicated an interest in shared care for diabetic retinopathy. 67% were interested in providing pre/post-operative cataract services. 61% were willing to become involved in shared care schemes for AMD. 47% indicated interest in expanding their role in paediatric services. |
| Baker et al. | 2016 | UK | During 2014-2015 | Glaucoma | Qualitative study of 189 patients, 25 community optometrists, 4 glaucoma specialist hospital optometrists, 5 ophthalmologists, 6 GPs and 4 commissioners using surveys, interviews and focus groups. | To explore views of all stakeholders regarding the  operation of community-based enhanced ophthalmic services | **Patients**: 99% (GRRS) and 100% (MECS) of patients were satisfied with the examination. 99% of MECS patients would recommend the service. 95% of participants in both schemes had confidence and trust in their optometrist **Optometrists** were enthusiastic about GRRS, feeling fortunate to practise in a ‘pro-optometry’ area. No major negatives were reported, although both schemes were limited to patient's resident within certain areas, and some inappropriate GP referrals occurred (MECS). Communication with hospitals was praised in GRRS but was variable, depending on hospital for MECS. Training for both schemes was valuable and appropriate but should be ongoing.  **GPs**: were very supportive, reporting the scheme would reduce secondary care referral numbers, although some MECS patients were referred back to GPs for medication.  **Ophthalmologists** expressed positive views and acknowledged that new care pathways would reduce unnecessary referrals and shorten patient waiting times.  **Commissioners** felt both schemes met or exceeded expectations in terms of quality of care and allowing patients to be seen quicker and more efficiently. |
| Konstantakopoulou et al. | 2016 | UK | September 2013-August 2014 | MECS | Retrospective, quantitative analysis of 2123 MECS appointments. Qualitative analysis of patient questionnaires. | To evaluate the clinical effectiveness, impact  on hospital attendances and patient satisfaction  with MECS | All patients (100%) (109/109) who completed the survey were satisfied with their visit to the optometrist and 99% would recommend the scheme to a friend;95% of the patients reported confidence and trust in their MECS optometrist and 90% were satisfied with the location they attended. |
| Konstantakopoulou et al. | 2014 | UK | _ | Glaucoma and MECS | Qualitative study of 43 optometrists, 6 ophthalmologists and 25 GPs using free-text questionnaires and telephone interviews. | To explore the views of optometrists, GPs and ophthalmologists regarding  community-based enhanced optometric services | **Optometrists**: Most common reason for participating in extended role programmes was for career development. Another reason for participation was the perceived benefit for patients and the wider NHS through improving pathways and enhancing glaucoma detection. 40% reported that participation was a means of receiving remuneration for services. Approximately 85% identified that training had a beneficial effect on their practice. Optometrists felt that MECS would improve communication with secondary eye care services. Non-participating optometrists believed that participating in the scheme would have required their practice to adapt significantly. **Ophthalmologists:** Ophthalmologists participated for reasons that were more patient centred: reduction of unnecessary referrals, relieving patient anxiety, improving patient care and reductions in patient waiting times **GPs:** Almost all GPs thought MECS would improve care and ‘journey’ for patients, as well as reduce waiting times. GPs believed that the scheme offers patients more choice and provides a cost effective and accessible service. |
| Syam et al. | 2010 | UK | February 2005-March 2007 | Glaucoma | Retrospective, quantitative analysis of 1184 glaucoma referrals and 72 patient satisfaction surveys. | To assess the role of specialist optometrists working in the community shared care for glaucoma patients. | **Patients**: 96% (69/72) of returned questionnaires indicated satisfaction with the scheme 9 patients expressed some confusion about the details of their follow-up appointment. |
| Sheen et al. | 2009 | UK | April-December 2006 | PEARS and WEHE | Prospective analysis of 6432 patients. Interviews with a subset of 289 patients. | To derive evidence for the efficacy of two optometric care services in Wales (PEARS and WEHE) | **Patients:** Of the 289 interviewees, 94.8%) were ‘‘very satisfied’’ and 15 (5.2%) ‘‘fairly satisfied’’ with the optometric service. 87.4% travelled less than 5 miles to an optometrist. |

**Supplementary Table 4**: Summary of studies focusing on the acceptability of enhanced referral refinement schemes.

| **Author(s)** | **Year** | **Location** | **Study Period** | **Study Design** | **Aim** | **Results** |
| --- | --- | --- | --- | --- | --- | --- |
| Bowes et al | 2018 | UK | April-December 2015 | Prospective, quantitative study of 712 direct referrals for cataract surgery | To report on defined key performance indicators (KPIs) of a cataract shared care scheme. | **Listing rates**: 591/712 patients (83%) were listed for cataract surgery at first visit. Of 449 GP routine clinical pathway referrals 282 patients (63%) were listed at first consultation. **Outcomes:** Of the 569 patients who had surgery(n=569), 402(71%) were discharged back to the community, 116 (20%) were followed up in a doctor-led clinic and 51 (9%) were followed up in a hospital optometrist led clinic. |
| Park et al. | 2009 | UK | March-May 2006 | A quantitative retrospective analysis of patients referred for cataract surgery (62 via optometric pathway and 62 via GP pathway) | To compare the quality of referrals and listing rates of direct optometric referrals vs traditional GP referrals for cataract surgery. | **Referral content**: Direct referrals were more likely to include information relating to objective visual loss (100 vs 87%, p= 0.0061) and to counsel the patient (97 vs 18%, p=0.0001). GP referrals were more likely to comment on personal circumstances (32 vs 3%, p=0.0001), past medical history (95 vs 68%, p=0.0001), and drug history (94 vs 69%, p= 0.0009).  **Operative rates:** Direct referrals had higher operative rates (87 vs 69%, p=0.0284). More patients from the traditional GP pathway were not listed, because the cataract was found to have no effect on their lifestyle (12 GP pathway, 4 direct pathway), or because the patient declined surgery (4 GP pathway, 2 direct pathway), or for other reasons (3 GP pathway, 2 direct pathway). |
| Lash et al. | 2006 | UK | 4th October- 6th December 2004  (2 months) | Quantitative prospective audit 351 optometrist referrals for cataract (162 GOS18 143 direct, 61 letters) | To review three types of optometrist referral (direct, GOS 18 and by letter) for information included and listing rates for surgery. | **Information included:** Full information was included in all direct referrals,10% (n=16) of GOS 18 referrals and 17% (n=8) of letter referrals.  **Listing rates:** The listing rates were 83%(n=119) for direct referrals, 78% (n=36) for letter referrals and 73% (n=117) for GOS18 referral p (chi-squared test P=0.087) |

**Supplementary Table 5**: Summary of studies focusing refinement schemes for cataract referrals.

| **Author(s)** | **Year** | **Location** | **Study Period** | **Study Design** | **Aim(s)** | **Condition(s)** | **Imaging used** | **Main Results** |
| --- | --- | --- | --- | --- | --- | --- | --- | --- |
| **Al Harby et al.** | 2022 | UK | June 2016- July 2017 | Prospective quantitative study of 400 patients attending naevus clinics. | To present the results of the NAEVUS study on a large prospective cohort to validate a virtual model for managing choroidal naevi referrals in terms of its safety | Naevo-melanocytic lesions | Wide-field colour imaging, auto-fluorescence imaging (AF), optical coherence tomography (OCT) and B-scan ultrasound | Agreement for management decisions between face-to-face and virtual pathways was 83.1%  (non-medical) and 82.6% (medical).  There were more over-referrals in the virtual pathway (non-medical 24.3%, medical 23.3% of gold standard discharge) and only two under-referrals (10.5% of gold standard referrals), both  borderline cases with minimal clinical risk.  The agreement for risk factors of growth (orange pigment, subretinal fluid, hyper-AF) ranged between 82.3% and 97.3% |
| **Hind et al.** | 2022 | UK | ? | Prospective quantitative study of 97 patients referred for suspect lid-lesions. | To assess the accuracy and feasibility of a pilot service by determining whether photograph-based assessment could be validated against a face-to-face clinic consultation | Eyelid lesions | External eye photographs | There was substantial agreement between diagnosis reached by clinicians reviewing patients F2F (Arm A) and clinicians reviewing photographs taken by a clinical photographer (Arm B (Ƙ = 0.72) and also between Arm A and clinicians reviewing photographs taken by a trained optometrist Arm C (Ƙ = 0.79) There were 10 lesions identified on F2F clinic review as suspected malignancy. All of these 10 lesions were also identified as suspicious by the clinicians reviewing the images from both Arm B and Arm C. There was substantial agreement in determining malignancy between Arm B and Arm A (Ƙ = 0.7) and almost perfect agreement between Arm C and Arm A (Ƙ = 1.0) 40% of patients were discharged without surgical intervention from the clinic. In Arm B, discharge was recommended in 51.6%, whereas in Arm C it was recommended in 28.4%. These differences were not statistically significant (Arm B vs A p = 0.145 and Arm C vs A 0.09). |
| **Muttuvelu et al.** | 2021 | Denmark | 1st August 2018-31st July 2019 | Quantitative retrospective analysis of 9938 referrals made to a web-based referral platform | To evaluate follow-up and referral patterns  after implementing a telemedical service for suspected posterior segment pathology | Posterior segment | Fundus photography | Mean time from routine referral to ophthalmologist review was 29 hours Mean time until optometrists communicated the review results to patients was 55 hours The average non-acute patient journey time was 115 hours,18 minutes  19.5% (n=1938) of the patients were referred onwards to the Danish national eye service. 14.4% (n=1431) of the referrals in did not need any further follow-up. 66.1% (n=6569) needed follow-up either by the optometrist (46.8% (4651 patients)) or within the TS (19.3% (n=1918)) |
| **Kern et al.** | 2020 | UK | April 2018-January 2019 | Quantitative retrospective analysis of 103 patients referred using a web-based referral platform | To report the implementation and initial results of a cloud-based referral platform to the HES | Retinal | Fundus photograph and OCT scan | 54 (52%) of the patients classified into the referral pathway did not require specialist referral  14 (14%) patients were reviewed as urgent and 35 patients (34%) as routine. For 7 (7%) patients, a diagnosis could not be made on clinical history and OCT scans alone The mean overall time for optometrists was 9.2min per patient The mean review time for referral refinement by an ophthalmologist was 3.0min in total |
| **Kortuem et al.** | 2018 | UK | September 2016-May 2017 | Quantitative retrospective analysis of 186 patient referrals | To report on the implementation and integration of virtual medical retina clinics | Retinal | Fundus photographs and OCT scan | The average waiting time for was 45.3 days (SD +/- 27.6 days)  46.8% of patients were reviewed for diabetic eye disease followed by dry AMD (10.2%) 45.5% of patients were discharged at first visit. 37.1% had virtual follow up and 17.4% required a F2F appointment.  The most common reason for a referral to a face-to-face clinic was poor image quality. |
| **Kotecha et al.** | 2017 | UK | 1st March 2014-31st March 2016 | Quantitative retrospective analysis of 1380 patients attending a virtual glaucoma clinic | To describe the outcomes of a technician-delivered glaucoma referral triaging service with virtual review data by a consultant ophthalmologist | Glaucoma | Stereo fundus imaging and anterior angle OCT | The average (SD) journey time in the clinic was 58 (16) min. The average (SD) time from patient attendance to consultant virtual review was 4 (4) days The number of patients discharged following virtual review was 855 (62%) 16 patients (1%) required same-day doctor assessment due to elevated IOP.  91 (6%) patients were booked for a follow-up in the glaucoma monitoring virtual clinic. 418 patients were referred for face-to-face outpatient review. 66/82 patients reviewed to assess false negative rate were discharged following consultation, equating to a false-negative rate of 20%. |
| **Balaskas et al.** | 2016 | UK | October 2014-March 2015 | Retrospective quantitative analysis of results from 102 patients attending naevus clinics. | Pilot study to test the safety and validity of a one-stop virtual clinic model relying on allied health professionals assessing naevomelanocytic lesions | Naevo-melanocytic lesions | Wide-field colour imaging, auto-fluorescence imaging (AF), optical coherence tomography (OCT) and B-scan ultrasound | Agreement for management decisions between gold standard and grader was 96.1% (98/102) Agreement for management between gold standard and ophthalmologist was 100% (102/102) Agreement in the rate of pick of erroneous referrals (i.e. nonchoroidal naevo-melanocytic lesions) between gold standard and masked grader was 98% (100/102) The agreement rate between masked ophthalmologist and masked grader was 94% for the presence of orange pigment detected on photographs, 97% for location of the lesion within one disc diameter of the optic disc, 93% for the presence of increased AF, 95% for increased AF attributable to drusen only or related to lipofuscin/subretinal fluid, 100% for the presence of subretinal fluid on OCT and 98% for the presence of choroidal elevation on OCT. |
| **El-Assal et al.** | 2015 | UK | June 2000–May 2006 (Group A) January 2007–December 2012 (Group B) | Quantitative retrospective audit of new HES glaucoma patient records. Group A (n=835)  and Group B (n=737) | To evaluate accuracy and outcome of community optometry referrals after implementation of the new 2006 GOS contract, the 2008 Eyecare Integration Programme pilot and the 2009 NICE guidelines. | Glaucoma | Optic disc photographs and visual field plots. | Waiting times reduced from 12.3(Group A) to 9.4 weeks (Group B). Significantly more patients kept first appointment (p = 0.0002) in group B.  At the first hospital appointment 633 eyes (37.6 %) were found to be normal in group A compared to 380 eyes (24.1 %) in group B.  There were significantly fewer normal patients (p < 0,0001), more glaucoma suspects (p < 0.0001), more open angle glaucoma patients (p = 0.0006) and fewer other conditions (p = 0.0024) in group B, compared to group A. |
| **Goudie et al.** | 2014 | UK (Scotland) | September 2010 - January 2011 | Quantitative, retrospective analysis of 358 e-referrals with attached digital images. | To quantify the effect of attaching digital images  to ophthalmic referrals. | All ocular conditions | Fundus photographs | All 358 images were of a quality that could be used to influence clinical decision making 53 referrals (18%) were deemed ‘urgent’ and were seen within 24–60 h 122 referrals (34%) did not result in an appointment with the HES, with 95 (25% of total) resulting in an ‘e-diagnoses.  2/254 patients (0.8%) who were given an appointment ‘did not attend’ |
| **Borooah et al.** | 2013 | UK (Scotland) | May 2006-April 2007 (Traditional referrals) May 2008-April 2009 (COERU) | Quantitative prospective analysis of 8821 referrals made using  a traditional referral pathway and 8707 referred  using an e-referral pathway | To assess a centralised ophthalmic electronic referral  unit (COERU) | All ocular conditions | Photographs | Waiting times reduced from a median of 14 weeks (0-32) with traditional referral to 4 weeks (0-12) with the COERU. No significant increase in no. of referrals (8821 vs 8707, p=0.38). Significantly less new patients seen face-to-face (8714 Vs 7462, p<0.0001) Significantly less unscheduled patients attending eye casualty (2671 Vs 1984, p<0.0001) Significantly less patients not attending scheduled appointments (645 Vs 503, p<0.0001) The departmental complaint rate reduced from 7.5 to 3.5 per annum with none relating to the COERU. There were no reported adverse events. |
| **Trikha et al.** | 2012 | UK | _ | Quantitative retrospective analysis of 100 general referrals, | To evaluate the Portsmouth glaucoma scheme, utilising virtual clinics. | Glaucoma | Optic disc images | 76% of 100 general referrals were deemed suitable for the refinement scheme. Optic disc assessment was gradable from the photographs 71% of the time. 11% of referrals into virtual clinic were subsequently given an appointment in the HES glaucoma clinic.  The positive predictive rate was 0.78 (95% CI 0.65–0.87). |
| **Kelly et al.** | 2011 | UK | June 2010-August 2011 | Quantitative analysis of 50 e-referrals for macular disease | To complete a service review of an e-referral system | Retinal pathology | Photographs | 96% of cases reviewed by an ophthalmologist within the next calendar day 34% of cases did not require onward referral and were followed up in primary care optometry  Anecdotal good image quality and accepted by patients. |
| **Cameron et al.** | 2009 | UK(Scotland) | July 2005- January 2007 | Quantitative prospective analysis of 346 e-referrals into an  e-referral system | To assess a pilot electronic referral system | All ocular conditions | Photographs | 160/346 (73%) referrals had ocular imaging attached. All of which were sufficient quality 60/346 (20%) of all referrals were for suspect macular disease 128/346 referrals deemed not to need hospital review of which there was 124/128 agreement at F2F appointment  3/114 patients contacted said they preferred F2F review over virtual but "the rest were extremely positive about the new referral pathway" |
| **Hanson et al.** | 2008 | Canada | 1st June 2004- 31st May 2006 | Quantitative, retrospective review of 171 patients (190 visits) | To report long-term results of a teleophthalmology triage service for optometry referrals. | Retinal pathology | Stereo fundus imaging | **Outcomes:** 7 patients (4.1%) were found to have no evidence of ocular pathology in either eye. The most common retinal abnormality identified was macular degeneration (123 eyes) and diabetic retinopathy (47 eyes). 82 patients (48%) did not require referral, whereas 89 patients (52.0%) were referred for clinical examination. 28 patients were referred back to the optometrist for follow-up following clinical assessment. **Image quality:** 53/76 (70%) of patients referred for clinical examination of suspect macular degeneration did not have photographs of sufficient quality to make a definitive diagnosis of the wet or dry form on their digital retinal examination **Patient travel:** There was a total travel savings of 24,413.99 km and 295.09 hours, and an average travel savings of 301.41 km and 3.64 hours, for those patients who could be assessed by teleophthalmology alone. |

**Supplementary Table 6**: Summary of studies focusing on asynchronous teleophthalmology outcomes.

| **Author(s)** | **Year** | **Location** | **Study Period** | **Referral Refinement** | **Asynchronous Review** | **Results** |
| --- | --- | --- | --- | --- | --- | --- |
| **Ford et al.** | 2019 | UK | January–October 2017 | Glaucoma | Consultant virtually reviewed fundus photographs, visual fields and clinical information for all patients seen. | For cases where the optometrist’s recommendation was changed, 7.6% required more urgent care, and 13% less. Numbers of patients discharged did not change. |
| **Keenan et al.** | 2015 | UK | 1st April 2010- 31st March 2013 | Glaucoma | Fundus photographs and visual fields  for each patient were sent via secure NHS email. Clinical information was uploaded. | Following virtual review, a further 5.7% (n= 99) patients were discharged. 3.6% of all patients (n= 62) who had been discharged following community OSI assessment were recalled to a consultant-led clinic. |
| **Ratnarajan et al.** | 2015 | UK | _ | Glaucoma | Assessment of (non-stereoscopic) optic disc photographs of 34 patients discharged from a glaucoma referral refinement scheme. | On virtual review by a consultant ophthalmologist, 13/34 (38%) were suspicious of glaucoma and 21 (62%) normal. Virtual review by consultant gave a sensitivity of 80% and specificity of 69% compared to the clinic-based assessment.  Virtual review by hospital optometrist gave a sensitivity of 80% and specificity of 97% compared to the clinic-based assessment. |
| **Roberts et al.** | 2015 | UK | February 2005-February 2009 | Glaucoma | Consultant virtually reviewed fundus photographs, visual fields and clinical information for all patients seen by level 1 SOGs and patients requested to be reviewed by level 2 SOGs | 971 (29.6%) were un-assessable mainly due to cataract or other media opacity. Level 2 SOGs had an 87.8% agreement/non-significant disagreement with the consultant. Level 1 SOGs had a 75.9% agreement/non-significant disagreement with the consultant. |
| **Devarajan et al.** | 2011 | UK | 4-year period | Glaucoma | Consultant reviewed disc photographs and completed referral refinement forms for 100 discharged patients | 98/100-disc images considered gradable 2/98 (2%) required follow up in the HES but neither were started on treatment False negative rate of 3-10% |
| **Syam et al.** | 2010 | UK | February 2005-March 2007 | Glaucoma | Review of all patients by the project lead using disc photographs | 360/2368 (15.2%) were unusable due to cataract Unusable visual fields were very small (0.5%) A significant disagreement between the project lead’s appraisal and findings of the SOGs was observed in: optic nerve morphology (11%), visual field (7%), diagnosis (12%), treatment (10%), and follow-up (17%) |

**Supplementary Table 7**: Summary of studies focusing on glaucoma referral refinement schemes combined with asynchronous teleophthalmology.

| **Author(s)** | **Year** | **Location** | **Study Period** | **Study Design** | **Aim(s)** | **Condition** | **Intervention** | **Main Results** |
| --- | --- | --- | --- | --- | --- | --- | --- | --- |
| **Stewart et al.** | 2022 | USA | February 2016-April 2018 | Prospective analysis of agreement | Study agreement between telemedicine and in-person examinations for diagnosing and managing patients. | Paediatric eye conditions | Synchronous teleophthalmology using Polycom video conferencing system Pivot head glasses, Topcon digital slit lamp with camera attachment and a Keeler Digital Wireless Indirect Ophthalmoscope | 210 patients were examined. 94 were comprehensive (new referral) and 116 were consultation (seen previously by attending optometrist) examinations. No primary diagnoses were changed between the telemedicine and in-person examinations. 2 non-primary diagnoses were changes but no management plans. 78.4% (consultation group) and 55.3% (comprehensive group) warranted being seen by a paediatric ophthalmologist. The remaining patients either did not need to be seen at all or could have been seen by a qualified paediatric optometrist.  In all examinations, the ophthalmologist was able to hear and see the patient and visualise areas of interest.  98.5% of parents felt comfortable with the quality of the telemedicine examination. 97.1% reported they would participate in another one in the future. |
| **Ghazala et al.** | 2021 | UK (Scotland) | Pre lockdown = 1st Match 2019-22nd March 2020  During lockdown = 23rd March 2020-30th April 2020 | Retrospective, analysis using a convenience sample from 154 responses from a survey of ophthalmologists. | To compare the uptake and two outcomes (avoided escalations to secondary care and conditions where escalation was or was not avoided) of live teleophthalmology before and after COVID-19 lockdown. | All ocular conditions | Synchronous teleophthalmology using a video slit lamp or an iPad Air 2 with a bespoke mount. | 134 calls were made pre-lockdown and 116 during-lockdown.  50/78 (64.1%) surveyed pre-lockdown said a referral to secondary care had been avoided versus 65/76 (85.5%) surveyed during-lockdown (p=0.001). Sub-speciality where escalation was avoided (n = 115) was predominantly anterior or posterior segment (n = 101). There were no differences in sub-speciality pre- and during lockdown: anterior segment 25/50 vs 35/65 (p = 0.34); posterior segment 16/ 50 vs 25/65 (p = 0.24). Lid, peri-orbital, neuro-ophthalmology and uveitis presentations formed a relatively greater proportion of cases where escalation was not avoided than the same conditions where escalation was avoided (n = 12/39 vs 14/115, p = 0.004) |
| **Ghazala et al.** | 2021 | UK (Scotland) | 23rd March- 16th June 2020 | Survey of experience of 6 referrals | To share a method of appropriately connecting patients directly to tertiary ophthalmology  centres where sub-specialist vitreoretinal (VR) surgical management is reduced. | VR referrals | Live teleophthalmology with a VR surgeon via a video or adapted slit lamp. | In 5/5 referrals for suspect RD, patients were listed directly for operation and avoided having to attend the local ophthalmology department. The mean Likert score for satisfaction with the teleophthalmology consultation was 5/5 from optometrists, ophthalmologists and patients. Optometrists, ophthalmologists and patients all gave a mean Likert score of 5/5 for likeliness to recommend this type of consultation to a friend, family member and/or colleague. Ophthalmologists gave a mean Likert score of 5/5 for sound quality, video quality and connection reliability. Optometrists gave a mean Likert score of 4.6/5 for sound quality, 4.6/5 for video quality and 5/5 connection reliability |
| **Kanabar et al.** | 2021 | UK | Primary care: 1st June-31st July 2020  Secondary care: 17th June-11th August 2020 | Quantitative retrospective and prospective analysis  of referrals. | The aim was to evaluate the COVID-19 urgent  eye care service (CUES) for primary and secondary care activity. | Urgent referrals | Primary care optometry telephone triage and HES emergency hotline. | 91.1-91.7% were initially deemed eligible for a telemedicine appointment. 53.3-55.6% were given face-to-face appointments following a telemedicine appointment. 13.0-14.3% of cases were eventually provisionally referred to secondary care HES. Of the 101 provisional referrals to MREH from CUES received, 69 (68.3%) were accepted  Of the 61 accepted referrals graded by the hospital clinicians, 39 (63.9%) were categorised as either being in ‘agreement’ or ‘partial agreement’. Of the 32 rejected referrals, 25 (78.1%) were rejected due to the condition not being deemed an emergency 420 telephone calls were recorded and signposted to either CUES, the MREH EED, or local hospitals/optometrist practices. In 56.0% (235 phone calls) the patient was advised to attend MREH EED and in 32.4% (136 phone calls) the patient was advised to see a CUES optometrist in the community. |
| **Moussa et al.** | 2020 | UK | Pre-Intervention January-February 2020  Post-intervention April-July 2020 | Quantitative retrospective audit of pre-intervention (n=2868) and post-intervention (n=4870) patient interactions | To examine the impact of a restructured  ophthalmic referral at a tertiary referral centre. | All ocular conditions | Telephone triage service and a new on-call phone triage system. An NHS.net e-referral system for use by community optometrist | Pre-intervention, 1281(44.7%) patients required face-to-face follow up compared to 1192 (24.5%) post-intervention (p<0.0001) There was a higher proportion of discharges (p<0.0001), reduction in face-to-face visits (p<0.0001) and reduction in patients discharged without requiring face-to-face consultations (p<0.0001) post-intervention. Comparing face-to-face appointments only, there was no significant change in discharge rate (p=0.7245) July 2020 (relaxed lockdown rules) had significantly fewer face-to-face appointments (p<0.0001) and a higher overall discharge rate (p=0.0006) compared to pre-intervention. |

**Supplementary Table 8**: Summary of studies focusing on synchronous teleophthalmology outcomes.
